# P2Y_12_ Receptor Blockade Augments Glycoprotein IIb‐IIIa Antagonist Inhibition of Platelet Activation, Aggregation, and Procoagulant Activity

**DOI:** 10.1161/JAHA.113.000026

**Published:** 2013-06-21

**Authors:** Michelle A. Berny‐Lang, Joseph A. Jakubowski, Atsuhiro Sugidachi, Marc R. Barnard, Alan D. Michelson, Andrew L. Frelinger

**Affiliations:** 1 Center for Platelet Research Studies, Division of Hematology/Oncology, Boston Children's Hospital, Dana‐Farber Cancer Institute, Harvard Medical School, Boston, MA, Japan (M.A.B.L., M.R.B., A.D.M., A.L.F.); 2 Lilly Research Laboratories, Eli Lilly and Company, Indianapolis, IN, Japan (J.A.J.); 3 Biological Research Laboratories, Daiichi Sankyo Co., Ltd, Tokyo, Japan (A.S.)

**Keywords:** glycoprotein IIb‐IIIa receptor antagonists, platelets, prasugrel

## Abstract

**Background:**

New antiplatelet agents that provide greater, more consistent inhibition of the platelet ADP receptor P2Y_12_ may be used in combination with glycoprotein (GP) IIb‐IIIa antagonists, but their combined effect on platelet function and procoagulant activity is not well studied. Therefore, the objective of this study was to evaluate the independent and complementary effects of P2Y_12_ and GPIIb‐IIIa inhibition on platelet function and procoagulant activity.

**Methods and Results:**

Healthy donor blood was treated with the active metabolite of prasugrel (R‐138727 5 μmol/L), GPIIb‐IIIa antagonists (abciximab 3 μg/mL or eptifibatide 0.9 μg/mL), and combinations thereof, exposed to physiologically relevant agonists (collagen and ADP) and then evaluated for markers of platelet activation and procoagulant activity. Significant interactions between R‐138727 and GPIIb‐IIIa antagonists were observed. R‐138727 and the GPIIb‐IIIa antagonists had additive inhibitory effects on collagen‐stimulated platelet aggregation and on the collagen plus ADP–stimulated level of activated platelet surface GPIIb‐IIIa. R‐138727 and abciximab each inhibited collagen plus ADP–stimulated platelet phosphatidylserine expression and prothrombin cleavage, and the combination produced greater inhibition than achieved with abciximab alone. In contrast, eptifibatide did not inhibit, but instead enhanced, collagen plus ADP–stimulated prothrombin cleavage. Addition of R‐138727 reduced prothrombin cleavage in eptifibatide‐treated samples, suggesting a novel mechanism for potential benefit from combined prasugrel and eptifibatide treatment.

**Conclusions:**

The complementary effects of abciximab and R‐138727 on platelet activation, aggregation, and procoagulant activity suggest their combined use may, to a greater degree than with either agent alone, reduce thrombus formation in vivo.

## Introduction

In addition to the most commonly used antiplatelet agent, aspirin, which inhibits platelet cyclooxygenase‐1, two distinct classes of antiplatelet agents with distinct mechanisms of action, glycoprotein (GP) IIb‐IIIa antagonists (eg, abciximab, eptifibatide) and antagonists of the platelet ADP receptor P2Y_12_ (eg, clopidogrel, prasugrel), are used for the prevention of cardiac ischemic complications in the setting of percutaneous coronary intervention (PCI).^1–2^ Abciximab is the Fab fragment of the chimeric human–murine monoclonal antibody 7E3, whereas eptifibatide is a cyclic hexapeptide, small‐molecule inhibitor.^3^ Abciximab and eptifibatide bind to the GPIIb‐IIIa (integrin α_IIb_β_3_) receptor of human platelets, inhibiting the final common pathway of platelet aggregation in response to all agonists. Clopidogrel and prasugrel are thienopyridine prodrugs, each with an active metabolite that irreversibly inhibits the platelet ADP receptor P2Y_12_ and, thereby, inhibits ADP‐induced platelet activation and aggregation.^1^

While aspirin plus clopidogrel is the most frequently orally administered antiplatelet combination in PCI,^4^ novel P2Y_12_ inhibitors such as ticagrelor and prasugrel are also used in the setting of acute coronary syndrome PCI.^5^ As GPIIb‐IIIa and P2Y_12_ antagonists target distinct steps of the thrombus formation process, supplementing aspirin plus P2Y_12_ antagonist therapy with a GPIIb‐IIIa antagonist enhances platelet inhibition and may have clinical benefits in patients, particularly patients with large thrombus burden or poor response to oral antiplatelet agents.^2,4,6^ Although combination GPIIb‐IIIa and P2Y_12_ inhibition on an aspirin background is used in the clinical setting, the combined effect of these inhibitors on platelet responses has not been well studied, particularly in the case of the newer P2Y_12_ agents.

In in vitro studies, the active metabolite of prasugrel, R‐138727, in the absence or presence of aspirin, inhibits platelet activation as well as platelet procoagulant and proinflammatory responses;^7–9^ however, the effect of concomitant prasugrel and GPIIb‐IIIa antagonist treatment on these end points remains unknown. Therefore, the objective of this study was to evaluate the independent and complementary effects of P2Y_12_ and GPIIb‐IIIa inhibition on platelet function. On a background of aspirin, we evaluated the combined effect of in vitro treatment of whole blood with the active metabolite of prasugrel, R‐138727, plus abciximab or eptifibatide on platelet activation, aggregation, and procoagulant activity.

## Methods

### Materials

ADP was purchased from Bio/Data Corporation, and fibrillar type I collagen was purchased from Chrono‐log. Gly‐Pro‐Arg‐Pro (GPRP) was purchased from Bachem. The fluorogenic activated factor XIII (FXIIIa) substrate was from Zedira. Fluorescein isothiocyanate (FITC)‐conjugated annexin V, FITC‐labeled PAC1 (an IgM monoclonal antibody [mAb] specific for the activated conformation of GPIIb‐IIIa), phycoerythrin (PE)‐Cy5–conjugated CD42b‐specific mAb, and PE‐conjugated CD42a‐ and CD62P‐specific mAbs were from Becton Dickinson. PE‐Cy5–labeled CD14‐specific mAb was from Beckman Coulter. FITC‐conjugated anti–tissue factor mAb (clone VD8) was from American Diagnostica. Purified human coagulation factors Va and Xa (FVa and FXa, respectively) were purchased from Haematologic Technologies. The prothrombin fragment 1.2 (F1.2) ELISA was from Dade Behring.

### Blood Collection and Treatment

Institutional review board–approved written informed consent was obtained from all subjects. Two hours after healthy volunteers ingested aspirin 325 mg (Bayer HealthCare), blood was collected into a final concentration of sodium citrate 0.32%, hirudin 25 μg/mL, or d‐Phe‐Pro‐Arg‐chloromethylketone 300 μmol/L (PPACK; EMD Biosciences). Platelet counts for all donors were within the normal range of 150 to 400×10^3^/μL. R‐138727 (provided by Daiichi Sankyo Company Ltd, Tokyo, Japan) was dissolved in DMSO and kept at −80°C in sealed vials. Immediately before use, R‐138727 was diluted in HEPES 10 mmol/L, NaCl 0.15 mol/L, pH 7.4 (HEPES‐saline). For all subsequent assays, whole blood was incubated for 30 minutes at 37°C with R‐138727 5 μmol/L, abciximab 3.0 μg/mL (ReoPro; Centocor), eptifibatide 0.9 μg/mL (Integrilin; Millennium Pharmaceuticals), R‐138727 5 μmol/L plus abciximab 3.0 μg/mL, R‐138727 5 μmol/L plus eptifibatide 0.9 μg/mL, or vehicle (DMSO diluted identically to R‐138727). R‐138727, abciximab, and eptifibatide concentrations used for these experiments were selected to correspond with the level of platelet inhibition achieved in patients treated with the current recommended dosing regimens: prasugrel at 60 mg loading dose and 10 mg/day maintenance dose;^10^ abciximab at 0.25 mg/kg intravenous bolus followed by continuous infusion of 0.125 μg/kg per minute;^11^ eptifibatide at 180 μg/kg intravenous bolus followed immediately by a continuous infusion of 2.0 μg/kg per minute and a second eptifibatide bolus of 180 μg/kg administered 10 minutes later.^12^ Concentrations of R‐138727, abciximab, and eptifibatide were calculated with respect to the plasma compartment of the blood to account for differences in hematocrit between donors. For selected experiments, platelet‐rich plasma (PRP) was prepared by centrifugation of treated whole blood for 10 minutes at 150*g*. For each assay subsequently described, 6 independent experiments were performed using blood collected from 6 different donors.

### Platelet Aggregometry

Light transmission platelet aggregation was evaluated in a Chrono‐log aggregometer in PPACK‐anticoagulated PRP in response to ADP 20 μmol/L or collagen 20 μg/mL. The aggregation response was recorded for a total of 6 minutes using Aggro/Link software (Chrono‐log). Platelet counts were not adjusted before the use of PRP in aggregation.

### Platelet Surface Expression of Activated GPIIb‐IIIa and P‐selectin

PPACK‐anticoagulated whole blood was incubated with FITC‐PAC1, PE anti‐CD62P, and PE‐Cy5 anti‐CD42a (as a platelet identifier) and either collagen 20 μg/mL plus ADP 20 μmol/L or no agonist (HEPES‐Tyrode's buffer) for 15 minutes at room temperature. Samples were then fixed by addition of 1% formaldehyde in HEPES‐saline. Flow cytometric analysis was performed in a calibrated Becton Dickinson FACSCalibur as previously described.^13^

### Platelet Surface Binding of Annexin V

PPACK‐anticoagulated whole blood was diluted 1:10 in HEPES‐Tyrode's buffer and incubated with collagen 20 μg/mL plus ADP 20 μmol/L or no agonist (HEPES‐Tyrode's buffer) for 20 minutes at 37°C. Phosphatidylserine expression on the platelet surface was determined by annexin V binding as previously described.^8^ Briefly, after incubation with collagen plus ADP or buffer, samples were mixed with FITC‐conjugated annexin V and a PE‐Cy5 anti‐CD42a antibody (as a platelet identifier) in the presence of CaCl_2_ 4 mmol/L, incubated for 15 minutes at room temperature, and fixed with 1% formaldehyde in HEPES‐saline. Flow cytometric analysis was performed in a calibrated Becton Dickinson FACSCalibur.

### Tissue Factor Expression on Monocyte–Platelet Aggregates and Single Platelets

The presence of monocyte–platelet aggregates and the expression of tissue factor on monocyte–platelet aggregates and single platelets were detected, as previously described,^14^ in PPACK‐anticoagulated whole blood stimulated with collagen 20 μg/mL plus ADP 20 μmol/L or no agonist (HEPES‐saline). In brief, whole blood was incubated with agonists for 15 minutes at room temperature and then incubated with a mixture of PE anti‐CD42a 1 μg/mL for platelet identification, PE‐Cy5 anti‐CD14 for monocyte identification, and FITC‐conjugated anti–tissue factor mAb 10 μg/mL for 20 minutes at room temperature. After incubation, FACS Lysing solution (Becton Dickinson) was added to samples, and flow cytometric analysis was performed in a Becton Dickinson FACSCalibur.

### Thrombin Generation

Hirudin‐anticoagulated whole blood was mixed for 15 minutes at 37°C with shaking with FXa 600 pmol/L and FVa 300 pmol/L combined with either collagen 20 μg/mL plus ADP 20 μmol/L or no agonist (HEPES‐Tyrode's buffer) in a 96‐well microtiter plate (total volume 100 μL, all concentrations are final concentrations in whole blood). The assay was stopped by the addition of 260 μL of EDTA 2 mmol/L in HEPES‐saline. Samples were centrifuged and thrombin generation was measured by the formation of F1.2 in the supernatant using the Enzygnost F1.2 ELISA.

### Activated FXIII Generation

For FXIII assays, blood was collected from healthy volunteers into sodium citrate, 24 hours after ingestion of aspirin 325 mg. As described earlier, blood was treated with R‐138727, abciximab, and eptifibatide, and PRP was prepared via centrifugation. Sodium citrate–anticoagulated PRP was combined with FXIIIa substrate solution (fluorogenic FXIIIa substrate 56 μmol/L, CaCl_2_ 20 mmol/L, FXa 60 pmol/L, FVa 30 pmol/L, and GPRP 10 mmol/L) and collagen 20 μg/mL plus ADP 20 μmol/L or no agonist (HEPES‐saline) in a 96‐well microtiter plate (all concentrations are final concentrations in PRP). Fluorescence was monitored (excitation at 313 nm and emission at 418 nm) in a microtiter plate reader for 45 minutes at 37°C with shaking. PPACK‐treated PRP was used as the plate blank.

### Statistical Methods

Data analyses were performed by the authors using GraphPad Prism Version 5.0 and VassarStats: Website for Statistical Computation.^15^ Data are presented as mean±SEM from 6 independent experiments. The effects of R‐138727, the GPIIb‐IIIa antagonists, and the interactions between R‐138727 and the GPIIb‐IIIa antagonists were determined by 2‐factor repeated measures ANOVA (RM‐ANOVA) with *P*<0.05 considered statistically significant. Posttests to compare individual treatment conditions assumed samples were from normal distributions and were by Student's paired *t* test or by 1‐sample *t* test (for comparison with a normalized baseline result). To account for multiple comparisons, only posttest *P* values <0.0071 (Bonferroni correction) were considered significant.

## Results

### Inhibition of Platelet Aggregation by P2Y_12_ and GPIIb‐IIIa Antagonists

**Figure 1.**
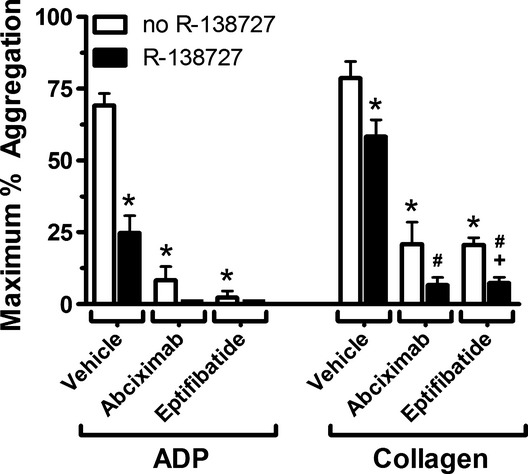
Effect of combined P2Y_12_ and GPIIb‐IIIa inhibition on ADP‐ or collagen‐induced platelet aggregation. Platelet‐rich plasma (PRP) was prepared from PPACK‐anticoagulated blood treated with vehicle or R‐138727 5 μmol/L in the absence or presence of abciximab 3 μg/mL or eptifibatide 0.9 μg/mL. PRP was stimulated with ADP 20 μmol/L or collagen 20 μg/mL and the extent of platelet aggregation was determined. Data are reported as mean± SEM from 6 experiments. *P<0.0071 vs no R‐138727, vehicle treatment; ^+^*P*<0.0071 vs corresponding treatment in the absence of R‐138727; ^#^*P*<0.0071 vs R‐138727, vehicle treatment. GP indicates glycoprotein; PPACK, d‐Phe‐Pro‐Arg‐chloromethylketone.

The P2Y_12_ antagonist R‐138727 has been previously shown to dose‐dependently inhibit ADP‐induced platelet aggregation.^7^ To investigate the combined effect of P2Y_12_ and GPIIb‐IIIa inhibition on a background of aspirin, platelet aggregation was studied in PRP from aspirin‐treated subjects, treated in vitro with R‐138727 alone or in combination with the GPIIb‐IIIa antagonists, abciximab or eptifibatide. Consistent with previous studies, when platelets were stimulated with ADP, aggregation was significantly inhibited in the presence of R‐138727 (Figure 1). Likewise, as expected, treatment with either GPIIb‐IIIa antagonist resulted in a marked decrease in ADP‐induced aggregation. However, the addition of either abciximab or eptifibatide to R‐138727 completely abrogated platelet aggregation (Figure 1). Two‐factor RM‐ANOVA of ADP‐induced platelet aggregation (Table) showed a significant effect of both R‐138727 (*P*<0.0001) and the GPIIb‐IIIa antagonists (*P*<0.0001) and a significant interaction between R‐138727 and the GPIIb‐IIIa antagonists (*P*=0.0009). When collagen was used as the agonist, the presence of R‐138727 resulted in a smaller, but still significant, decrease in maximal platelet aggregation (Figure 1). As with ADP‐induced aggregation, the GPIIb‐IIIa antagonists significantly inhibited collagen‐induced platelet aggregation compared with vehicle. Combined treatment with R‐138727 plus abciximab or eptifibatide resulted in a markedly reduced aggregation response compared with R‐138727 treatment alone (Figure 1). Furthermore, when R‐138727 was added to the GPIIb‐IIIa antagonists, platelet aggregation was decreased beyond the level of aggregation with GPIIb‐IIIa inhibition alone.

**Table 1.** Statistical analysis of platelet activation, aggregation, and procoagulant responses to ADP 20 μmol/L and collagen 20 μg/mL in the presence of R‐138727 5 μmol/L, abciximab 3 μg/mL, or eptifibatide 0.9 μg/mL, and combinations thereof

	Factor	*P* Value
Platelet aggregation—ADP (maximum % aggregation)	R‐138727	<0.0001
	GPIIb‐IIIa antagonists	<0.0001
	Interaction	0.0009
Platelet aggregation—collagen (maximum % aggregation)	R‐138727	0.0021
	GPIIb‐IIIa antagonists	<0.0001
	Interaction	0.3960
Activated GPIIb‐IIIa—collagen+ADP (PAC1 MFI)	R‐138727	<0.0001
	GPIIb‐IIIa antagonists	<0.0001
	Interaction	<0.0001
Platelet surface P‐selectin—collagen+ADP (MFI)	R‐138727	0.0012
	GPIIb‐IIIa antagonists	0.0131
	Interaction	0.0223
% MPAs—collagen+ADP	R‐138727	0.0008
	GPIIb‐IIIa antagonists	0.0014
	Interaction	0.0004
Platelet fluorescence in MPAs—collagen+ADP (MFI)	R‐138727	0.0006
	GPIIb‐IIIa antagonists	0.0002
	Interaction	0.0001
% Annexin V–positive platelets—collagen+ADP	R‐138727	0.0010
	GPIIb‐IIIa antagonists	<0.0001
	Interaction	<0.0001
MPA tissue factor—collagen+ADP (MFI)	R‐138727	0.0007
	GPIIb‐IIIa antagonists	<0.0001
	Interaction	0.0001
Platelet tissue factor—collagen+ADP (MFI)	R‐138727	<0.0001
	GPIIb‐IIIa antagonists	0.2341
	Interaction	0.0012
Plasma F1.2—collagen+ADP (normalized)	R‐138727	0.0002
	GPIIb‐IIIa antagonists	0.0102
	Interaction	0.0183
Relative FXIIIa activity—collagen+ADP (1/time to V_max_, normalized)	R‐138727	0.3542
	GPIIb‐IIIa antagonists	0.0136
	Interaction	0.8089

Two‐factor ANOVA with repeated measures on both factors. GP indicates glycoprotein; MFI, mean fluorescence intensity; MPA, monocyte–platelet aggregate; V_max_, maximum velocity.

### Platelet Surface Activated GPIIb‐IIIa and P‐selectin

**Figure 2.**
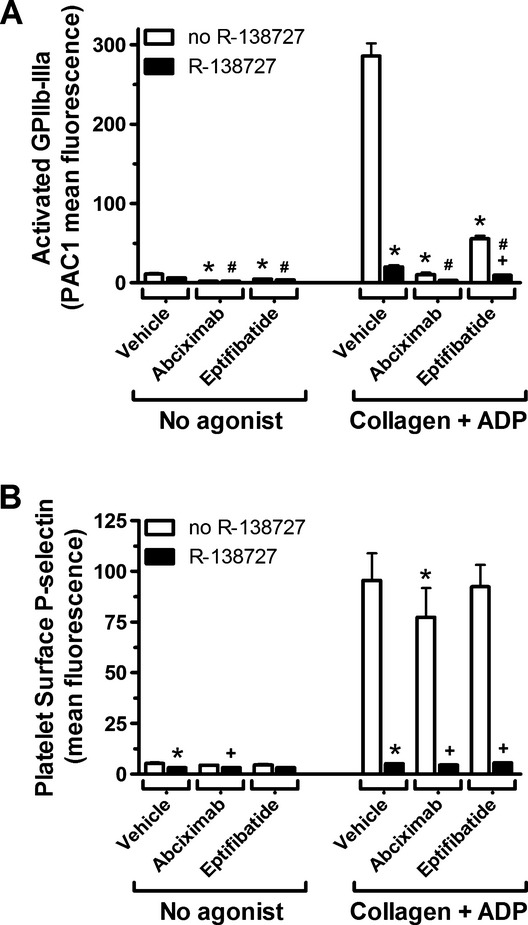
Inhibition of platelet surface expression of activated GPIIb‐IIIa and P‐selectin by R‐138727 plus abciximab or eptifibatide. PPACK‐anticoagulated whole blood treated with R‐138727, abciximab, or eptifibatide, and combinations thereof, was incubated with collagen 20 μg/mL plus ADP 20 μmol/L or no agonist and labeled with fluorescently‐conjugated antibodies for analysis by whole blood flow cytometry. A, Platelet surface activated GPIIb‐IIIa, as determined by platelet binding of the monoclonal antibody PAC1 (mean fluorescence intensity). B, Platelet surface expression of P‐selectin (mean fluorescence intensity). Data are shown as mean±SEM, n=6. *P<0.0071 vs no R‐138727, vehicle treatment; ^+^*P*<0.0071 vs corresponding treatment in the absence of R‐138727; ^#^*P*<0.0071 vs R‐138727, vehicle treatment. GP indicates glycoprotein; PPACK, d‐Phe‐Pro‐Arg‐chloromethylketone.

Because platelet aggregation is dependent on the activation of GPIIb‐IIIa,^16^ the combined effect of P2Y_12_ and GPIIb‐IIIa inhibition on the expression and availability of activated GPIIb‐IIIa was evaluated in whole blood treated with R‐138727 alone or in combination with abciximab or eptifibatide. After stimulation with collagen plus ADP, platelet surface activated GPIIb‐IIIa expression, as reflected by mAb PAC1 binding, was, as expected, markedly increased compared with unstimulated samples (Figure 2A). R‐138727 treatment caused a substantial reduction in the level of surface activated GPIIb‐IIIa. The presence of either GPIIb‐IIIa antagonist significantly reduced PAC1 binding to activated GPIIb‐IIIa in collagen plus ADP–stimulated samples (Figure 2A). PAC1 binding was further inhibited when R‐138727 was added to abciximab‐ or eptifibatide‐treated samples, and combined GPIIb‐IIIa and P2Y_12_ antagonism resulted in significant inhibition beyond R‐138727 treatment alone (Figure 2A). In unstimulated (no agonist) samples with low baseline activated GPIIb‐IIIa, PAC1 binding was reduced by either GPIIb‐IIIa inhibitor and, more significantly, inhibited by the combination of R‐138727 plus abciximab or eptifibatide (Figure 2A).

To further assess the level of platelet activation with combined P2Y_12_ and GPIIb‐IIIa inhibition, we evaluated the platelet surface expression of P‐selectin. Two‐factor RM‐ANOVA of collagen plus ADP–stimulated platelet surface P‐selectin (Table) showed a significant effect of R‐138727 (*P*=0.0012), the GPIIb‐IIIa antagonists (*P*=0.0131), and a significant interaction between R‐138727 and the GPIIb‐IIIa antagonists was present (*P*=0.0223). R‐138727 caused a large, significant decrease (≈95%, *P*=0.0011, paired *t* test) in platelet surface P‐selectin expression in collagen plus ADP–stimulated blood (Figure 2B); a smaller, significant decrease (≈19%, *P*=0.0067, paired *t* test) was observed with abciximab. No change in collagen plus ADP–stimulated P‐selectin expression was observed in the presence of eptifibatide. When abciximab or eptifibatide was used in combination with R‐138727, the decrease in P‐selectin expression was comparable to that observed with only R‐138727 treatment (Figure 2B).

### Monocyte–Platelet Aggregates

**Figure 3.**
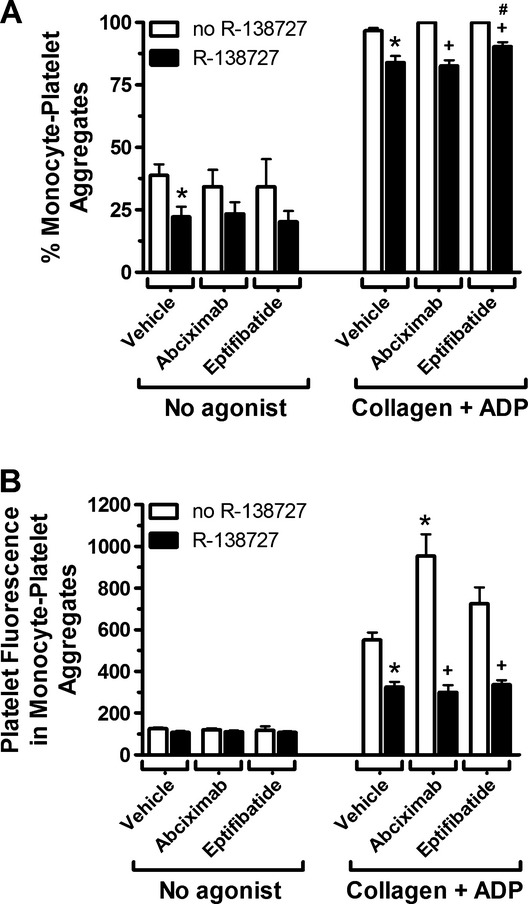
ADP plus collagen‐induced monocyte–platelet aggregates in the presence of P2Y_12_ and GPIIb‐IIIa antagonists. Whole blood anticoagulated with PPACK was stimulated with collagen 20 μg/mL plus ADP 20 μmol/L or no agonist, fluorescently‐labeled for monocytes and platelets, and analyzed flow cytometrically. A, Percentage of monocytes with platelet attached. B, Platelet fluorescence (mean fluorescence intensity) in monocyte–platelet aggregates. Results are mean±SEM (n=6). *P<0.0071 vs no R‐138727, vehicle treatment; ^+^*P*<0.0071 vs corresponding treatment in the absence of R‐138727; ^#^*P*<0.0071 vs R‐138727, vehicle treatment. GP indicates glycoprotein; PPACK, d‐Phe‐Pro‐Arg‐chloromethylketone.

As an additional marker of the level of platelet activation with combined P2Y_12_ and GPIIb‐IIIa inhibition, monocyte–platelet aggregates were measured with and without collagen plus ADP stimulation. In the absence of antiplatelet agents, as expected, collagen plus ADP increased the percentage of monocytes bound to platelets (monocyte–platelet aggregates) and the platelet fluorescence in monocyte–platelet aggregates (Figure 3A and 3B). By 2‐factor RM‐ANOVA, R‐138727, GPIIb‐IIIa antagonists, and the interaction between R‐138727 and GPIIb‐IIIa antagonists were highly significant for the collagen plus ADP–stimulated percentage of monocyte–platelet aggregates and the platelet fluorescence in monocyte–platelet aggregates (Table). In collagen plus ADP–stimulated samples, R‐138727 reduced the percentage of monocyte–platelet aggregates and the level of platelet fluorescence in the aggregates, indicating a reduced number of platelets in the aggregates (Figure 3). Although abciximab and eptifibatide each resulted in numerical increases in the percentage of monocyte–platelet aggregates and platelet fluorescence in monocyte–platelet aggregates, in posttests, only the abcximab‐induced increase in platelet fluorescence remained statistically significant. Addition of R‐138727 to abciximab abrogated this increase, reducing platelet fluorescence in monocyte–platelet aggregates to the level observed with R‐138727 treatment alone (Figure 3).

### Platelet Surface Expression of Phosphatidylserine

**Figure 4.**
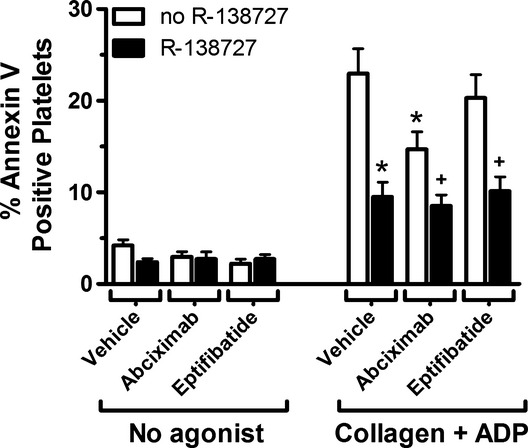
Effect of R‐138727 in combination with abciximab or eptifibatide on platelet surface phosphatidylserine exposure. Surface exposure of phosphatidylserine was determined flow cytometrically by annexin V binding of platelets from PPACK‐anticoagulated blood incubated with collagen 20 μg/mL plus ADP 20 μmol/L or no agonist. Data are shown as the percentage of platelets positive for annexin V staining, mean±SEM, n=6. *P<0.0071 vs no R‐138727, vehicle treatment; ^+^*P*<0.0071 vs corresponding treatment in the absence of R‐138727; ^#^*P*<0.0071 vs R‐138727, vehicle treatment. PPACK indicates d‐Phe‐Pro‐Arg‐chloromethylketone.

In addition to their role in aggregate formation, activated platelets promote coagulation through surface exposure of procoagulant phosphatidylserine.^17^ As expected, stimulation with collagen plus ADP increased the percentage of annexin V–positive platelets (Figure 4). Two‐factor RM‐ANOVA analysis (Table) showed a significant effect of both R‐138727 (*P*=0.0010) and the GPIIb‐IIIa antagonists (*P*<0.0001) and a significant interaction between R‐138727 and the GPIIb‐IIIa antagonists (*P*<0.0001). Individual treatment with R‐138727 or abciximab reduced annexin V binding in collagen plus ADP–stimulated samples (annexin V–positive platelets 22.9±2.7% with vehicle versus 9.5±1.6% for R‐138727, *P*=0.0004, and 14.7±1.9% for abciximab, *P*=0.0004), but no significant effect was observed with eptifibatide (*P*=0.0247 versus vehicle; nonsignificant with Bonferroni correction). No further inhibition was seen when abciximab or eptifibatide was added to R‐138727 treatment (Figure 4).

### Tissue Factor Expression on Monocyte–Platelet Aggregates and Individual Platelets

**Figure 5.**
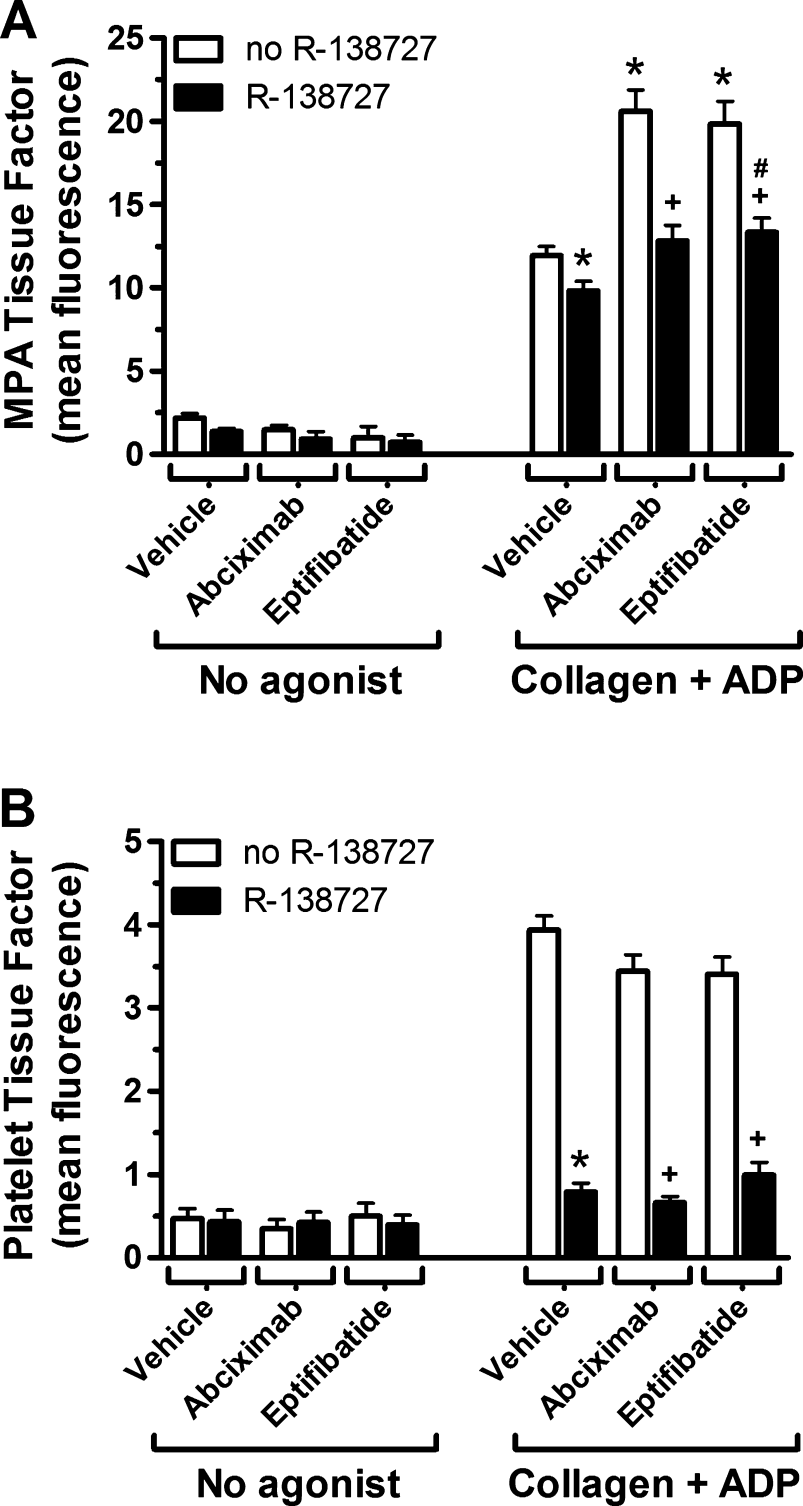
Tissue factor expression on monocyte–platelet aggregates (MPAs) and individual platelets in the presence of R‐138727 plus abciximab or eptifibatide. MPAs and individual platelets from PPACK‐anticoagulated blood stimulated with collagen 20 μg/mL plus ADP 20 μmol/L or no agonist were stained with an anti‐tissue factor antibody and analyzed flow cytometrically. A, Tissue factor on MPAs (fluorescence, arbitrary units). B, Tissue factor on individual platelets (fluorescence, arbitrary units). Results are reported as mean±SEM from 6 independent experiments. *P<0.0071 vs no R‐138727, vehicle treatment; ^+^*P*<0.0071 vs corresponding treatment in the absence of R‐138727; ^#^*P*<0.0071 vs R‐138727, vehicle treatment. PPACK indicates d‐Phe‐Pro‐Arg‐chloromethylketone.

Beyond platelet surface expression of phosphatidylserine, platelets and monocyte–platelet aggregates can stimulate coagulation through surface expression of tissue factor. In previous studies, treatment with R‐138727 decreased the level of tissue factor on monocyte–platelet aggregates.^8^ To extend these studies, we investigated the effect of R‐138727 combined with abciximab or eptifibatide on procoagulant surface expression of tissue factor. Stimulation with collagen plus ADP increased the level of tissue factor fluorescence on both monocyte–platelet aggregates and single platelets (Figure 5A and 5B). Two‐factor RM‐ANOVA analysis of tissue factor on monocyte–platelet aggregates (Table) showed significant effects of R‐138727 (*P*=0.0007), GPIIb‐IIIa antagonists (*P*<0.0001), and the interaction between R‐138727 and GPIIb‐IIIa antagonists (*P*=0.0001). Tissue factor on monocyte–platelet aggregates induced by collagen plus ADP was reduced by R‐138727 (Figure 5A). In parallel to the increase in monocyte–platelet aggregates observed with abciximab or eptifibatide (Figure 3), individual treatment with either GPIIb‐IIIa antagonist caused an increase in monocyte–platelet tissue factor (Figure 5A). The addition of R‐138727 to abciximab or eptifibatide reduced tissue factor levels on monocyte–platelet aggregates. When tissue factor on individual platelets stimulated with collagen plus ADP was examined, R‐138727 treatment caused significant inhibition, while the GPIIb‐IIIa antagonists had no significant effect (2‐factor RM‐ANOVA R‐138727, *P*<0.0001; GPIIb‐IIIa antagonists, *P*=0.2341; Table) on platelet tissue factor (Figure 5B).

### Platelet‐Dependent Coagulation Factor Activation: F1.2 Generation and FXIIIa Activity

**Figure 6.**
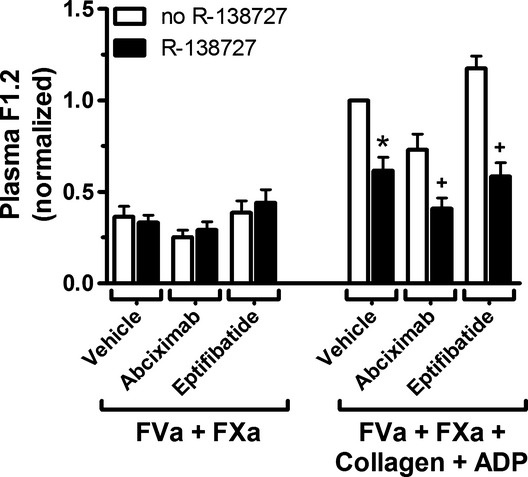
Inhibition of P2Y_12_ and GPIIb‐IIIa in platelet‐dependent thrombin generation. Hirudin‐anticoagulated blood was incubated with FXa 600 pmol/L and FVa 300 pmol/L with collagen 20 μg/mL plus ADP 20 μmol/L or with buffer. Thrombin generation was determined by the level of prothrombin fragment F1.2 as measured by ELISA. Results shown are normalized per subject to collagen plus ADP with no‐R138727, vehicle treatment; mean±SEM, n=6. *P<0.0071 vs no R‐138727, vehicle treatment; ^+^*P*<0.0071 vs corresponding treatment in the absence of R‐138727; ^#^*P*<0.0071 vs R‐138727, vehicle treatment. GP indicates glycoprotein; FVa and FXa, coagulation factors Va and Xa, respectively.

To determine the effects of combined platelet P2Y_12_ and GPIIb‐IIIa inhibition on coagulation, we investigated platelet‐dependent enhancement of thrombin generation and activation of FXIII in the presence of R‐138727 plus abciximab or eptifibatide. When thrombin generation was measured by the formation of F1.2 in whole blood, addition of collagen plus ADP, in the presence of priming concentrations of FVa and FXa, caused a marked increase in F1.2 compared with blood primed with FVa and FXa but without the addition of collagen plus ADP (Figure 6). Two‐factor RM‐ANOVA (Table) showed a significant effect of R‐138727 (*P*=0.0002) and the GPIIb‐IIIa antagonists (*P*=0.0102) and a significant interaction between R‐138727 and the GPIIb‐IIIa antagonists (*P*=0.0183). Collagen plus ADP–enhanced F1.2 levels were reduced by 38% by R‐138727 treatment compared with vehicle (*P*=0.0064, 1‐sample *t* test, Figure 6). Treatment with abciximab alone reduced the collagen plus ADP–dependent increase in F1.2 (*P*=0.0250, 1‐sample *t* test), while eptifibatide treatment enhanced collagen plus ADP–stimulated F1.2 (*P*=0.0482, 1‐sample *t* test), although neither effect reached statistical significance after Bonferroni correction. The combination of R‐138727 plus abciximab or eptifibatide reduced F1.2 compared with individual GPIIb‐IIIa antagonist treatment and resulted in a level of F1.2 that was comparable to R‐138727 treatment alone (Figure 6).

**Figure 7.**
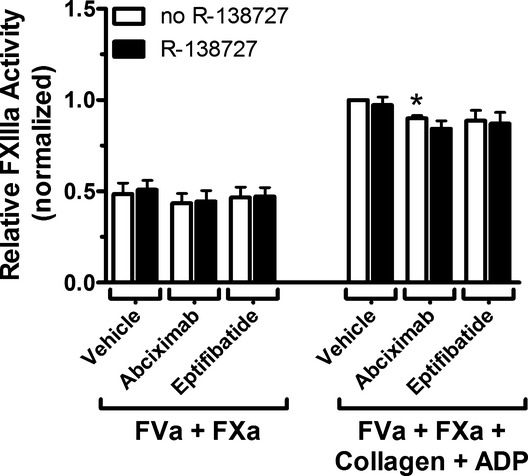
Platelet‐dependent FXIIIa generation in the presence of R‐138727 plus abciximab or eptifibatide. Sodium citrate–anticoagulated blood was incubated with solution of CaCl_2_, GPRP (to inhibit fibrin polymerization), FXa 60 pmol/L, and FVa 30 pmol/L combined with collagen 20 μg/mL plus ADP 20 μmol/L or no agonist. FXIIIa generation was monitored by the cleavage of a fluorogenic FXIIIa substrate, and the relative FXIIIa activity was reported as the reciprocal of the time to reach V_max_, normalized per subject by the value obtained for a vehicle‐treated sample stimulated with collagen plus ADP. Results are mean±SEM, from 6 experiments. *P<0.0071 vs no R‐138727, vehicle treatment; ^+^*P*<0.0071 vs corresponding treatment in the absence of R‐138727; ^#^*P*<0.0071 vs R‐138727, vehicle treatment. GPRP indicates Gly‐Pro‐Arg‐Pro; FVa and FXa, coagulation factors Va and Xa, respectively; V_max_, maximum velocity.

FXIIIa generation, primed by addition of FVa and FXa, was significantly increased by platelet activation with collagen plus ADP (Figure 7). Relative FXIIIa activity in collagen plus ADP–activated samples was not significantly affected by treatment with R‐138727 or eptifibatide alone (Figure 7). However, the relative FXIIIa activity was significantly, albeit modestly, decreased in the presence of abciximab (*P*=0.0015, 1‐sample *t* test, Figure 7).

## Discussion

The main findings of this study are as follows. (1) The combination of P2Y_12_ and GPIIb‐IIIa antagonists, at whole blood concentrations that demonstrate pharmacological efficacy, resulted in greater inhibition of ADP– and collagen–induced platelet aggregation and collagen plus ADP–induced platelet surface activated GPIIb‐IIIa than with either antagonist alone (Figures 1 and 2A). (2) The level of tissue factor on monocyte–platelet aggregates was increased by GPIIb‐IIIa inhibitors, and the increase was significantly reduced by the active metabolite of prasugrel, R‐138727 (Figure 5A). (3) R‐138727 and abciximab each significantly inhibited collagen plus ADP–stimulated platelet surface expression of phosphatidylserine (Figure 4) and reduced thrombin generation (Figure 6). In contrast, eptifibatide alone increased, albeit modestly, platelet activation–dependent thrombin generation (Figure 6). The combination of R‐138727 with eptifibatide reduced the level of thrombin generation to that seen with R‐138727 alone. (4) Abciximab, but not eptifibatide, reduced FXIIIa activation downstream from platelet activation with collagen plus ADP (Figure 7).

### Clinical Evidence Suggesting a Benefit From Combined GPIIb‐IIIa and P2Y_12_ Inhibition

The presently described additive effects of GPIIb‐IIIa antagonists and prasugrel's active metabolite on platelet activation, aggregation, and procoagulant activities have not previously been reported. While current indications for GPIIb‐IIIa antagonist use are limited,^3,18^ broader application of GPIIb‐IIIa antagonists in combination with P2Y_12_ antagonists may have clinical benefit. For example, when prasugrel was administered with or without the GPIIb‐IIIa antagonist tirofiban in ST‐segment elevation acute myocardial infarction patients on aspirin, platelet inhibition was suboptimal (as measured by ADP‐induced aggregation) for at least 2 hours with prasugrel alone, but the addition of tirofiban led to a significantly higher degree of platelet inhibition.^6^ Furthermore, a recent meta‐analysis of randomized trials of patients undergoing elective PCI with stents and periprocedural thienopyridines, assigned to randomly receive a GPIIb‐IIIa antagonist or control, show a clinical benefit of GPIIb‐IIIa inhibitors (both abciximab and small‐molecule inhibitors).^2^ Specifically, GPIIb‐IIIa antagonists added on a background of aspirin and a thienopyridine were shown to reduce nonfatal myocardial infarction, without an increase in major bleeding, but with an increase in minor bleeding. These studies combined with the present study suggest that GPIIb‐IIIa antagonists are beneficial in inhibiting platelet function when used in combination with P2Y_12_ antagonists.

### Augmented Inhibition of Platelet Activation, Aggregation, and Procoagulant Activities by Combined Use of R‐138727 and GPIIb‐IIIa Antagonists

Because light transmission platelet aggregation has been the de facto gold standard for platelet function testing throughout the development of the GPIIb‐IIIa antagonists and newer P2Y_12_ antagonists,^19^ the additive inhibitory effect of R‐138727 plus abciximab or eptifibatide on collagen– and ADP–stimulated platelet aggregation is not surprising. The additional inhibition of platelet aggregation and platelet surface activated GPIIb‐IIIa expression by combined P2Y_12_ and GPIIb‐IIIa antagonists may in part explain the clinical benefit resulting from their combined use in the meta‐analysis described earlier.^2^ Downstream effects of combined inhibition with abciximab and R‐138727 observed in the present study [reduced annexin V–positive platelets (Figure 4) and reduced conversion of prothrombin to thrombin with concomitant release of F1.2 (Figure 6)] are also likely to be mechanistically linked to the clinical benefit observed in the meta‐analysis.^2^ The results of the present study are similar to that reported for the combination of cangrelor, a reversible P2Y_12_ antagonist and abciximab or tirofiban, which resulted in additive inhibition of platelet aggregate formation, dense granule secretion, soluble CD40 ligand release, annexin V binding, and procoagulant microparticle formation, and to a study of in vivo clopidogrel plus in vitro GPIIb‐IIIa inhibition that demonstrated additive inhibition of ADP‐ or collagen‐induced platelet aggregation and fibrinogen binding to GPIIb‐IIIa.^20–21^

### Enhanced Procoagulant Status Following Treatment With GPIIb‐IIIa Antagonists Alone, and Ablation by R‐138727

The results of previous studies on the effect of GPIIb‐IIIa inhibitors on monocyte–platelet aggregate formation and tissue factor on monocyte–platelet aggregates are inconsistent, with GPIIb‐IIIa inhibitors reported to both augment and inhibit these processes.^22–25^ In agreement with the present results, separate studies have shown that abciximab enhanced ADP‐induced platelet fluorescence in monocyte–platelet aggregates,^22^ and eptifibatide enhanced tissue factor expression on monocytes.^23^ The enhanced procoagulant status observed after GPIIb‐IIIa antagonist treatment may be relevant to the increased thrombotic complications observed with oral GPIIb‐IIIa antagonists.^26^ In the current study, whole blood stimulated with collagen plus ADP in the presence of either abciximab or eptifibatide appears to be in a procoagulant state, as evidenced by increased exposure of tissue factor antigen on monocyte–platelet aggregates (Figure 5A), together with an increase in platelet mass associated with each monocyte (Figure 3B). Indeed, platelet activation–dependent prothrombinase activity is modestly enhanced in the presence of eptifibatide (Figure 6). However, abciximab under the same conditions reduced prothrombinase activity (Figure 6), thus demonstrating a difference in the ability of eptifibatide (a 1000 Da, small molecule, reversible GPIIb‐IIIa antagonist) and abciximab (a much larger [50 000 Da], tight‐binding GPIIb‐IIIa antagonist) to modulate platelet‐dependent procoagulant activity.

Prothrombin has been shown to bind to GPIIb‐IIIa and this binding accelerates thrombin generation.^27^ With its larger size, abciximab (and other antibody inhibitors) may not only block prothrombin binding to GPIIb‐IIIa but may also sterically hinder other binding reactions on the platelet surface that are important for thrombin generation, such as coagulation factor binding to surface‐exposed phosphatidylserine. This concept is in agreement with a previous study, showing that abciximab more strongly inhibited annexin V and factor V/Va binding to the platelet surface than eptifibatide.^28^ The molecular size difference and specific molecular interactions between each antagonist and GPIIb‐IIIa may explain the variable response in prothrombin activation. Because tissue factor activation is modulated by protein disulfide‐isomerase,^29^ it is also tempting to speculate that eptifibatide and abciximab differentially affect this activation.

In the present study, the addition of R‐138727 to GPIIb‐IIIa antagonists resulted in a significant decrease compared with GPIIb‐IIIa inhibitors alone in the percentage of monocyte–platelet aggregates, tissue factor expression on monocyte–platelet aggregates, and thrombin generation. This is in agreement with a previous study on clopidogrel and abciximab which reported that the addition of clopidogrel to abciximab treatment ablated the abciximab‐induced increase in monocyte–platelet aggregates.^22^ Taken together, the presently‐described ablation by prasugrel's active metabolite of the GPIIb‐IIIa antagonist‐induced increase of tissue factor on monocyte–platelet aggregates and the eptifibatide‐induced increase in thrombin generation, suggests that inhibition of the platelet P2Y_12_ ADP receptor may reduce the prothrombotic effects of GPIIb‐IIIa antagonists.

### Study Limitations and Strengths

This study used blood from healthy subjects rather than from acute coronary syndrome patients, the population for whom these drugs have an FDA‐approved indication, to minimize the variables of comedications and the degree of disease. However, all healthy donors were treated with aspirin to correspond to the clinical conditions where prasugrel and GPIIb‐IIIa antagonists might be coadministered. The power to detect treatment differences is limited by the small sample size (n=6); however, significant effects of GPIIb‐IIIa and/or P2Y_12_ inhibition were detected for each end point, even after application of the conservative Bonferroni correction.

## Conclusions

The presently described complementary effects of abciximab and prasugrel's active metabolite on collagen plus ADP–induced platelet activation, aggregation, and procoagulant activity suggest that the combined use of abciximab and prasugrel may, to a greater degree than with either agent alone, reduce and destabilize thrombus formation in vivo. The GPIIb‐IIIa antagonist‐induced increase of tissue factor on monocyte–platelet aggregates and the eptifibatide‐induced increase in thrombin generation were attenuated by prasugrel's active metabolite, suggesting a novel mechanism for reducing the prothrombotic effects of these GPIIb‐IIIa antagonists.
